# In Vitro Fossilization for High Spatial Resolution Quantification of Elements in Plant-Tissue Using LA-ICP-TOFMS

**DOI:** 10.1021/acs.analchem.3c05849

**Published:** 2024-03-14

**Authors:** Pascal Becker, Thomas Nauser, Matthias Wiggenhauser, Beat Aeschlimann, Emmanuel Frossard, Detlef Günther

**Affiliations:** † Laboratory of Inorganic Chemistry, Department of Chemistry and Applied Biosciences, ETH Zurich, Zurich 8093, Switzerland; ‡ Institute of Agricultural Sciences, ETH Zurich, Eschikon 33, Lindau CH-8315, Switzerland

## Abstract

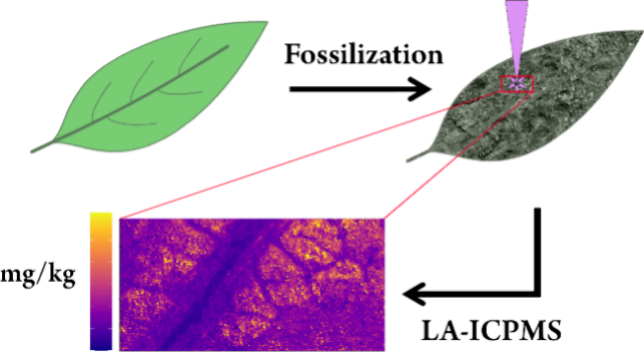

Laser ablation in combination with an inductively coupled plasma time-of-flight mass spectrometer (LA-ICP-TOFMS) is an upcoming method for rapid quantitative element mapping of various samples. While widespread in geological applications, quantification of elements in biotissues remains challenging. In this study, a proof-of-concept sample preparation method is presented in which plant-tissues are fossilized in order to solidify the complex biotissue matrix into a mineral-like matrix. This process enables quantification of elements by using silicone as an internal standard for normalization while also providing consistent ablation processes similar to minerals to reduce image blurring. Furthermore, it allows us to generate a quantitative image of the element composition at high spatial resolution. The feasibility of the approach is demonstrated on leaves of sunflowers (*Helianthus annuus*), soy beans (*Glycine max*), and corn (*Zea mays*) as representatives for common crops, which were grown on both nonspiked and cadmium-spiked agricultural soil. The quantitative results achieved during imaging were validated with digestion of whole leaves followed by ICP-OES analysis. LA-ICP-TOFMS element mapping of conventionally dried samples can provide misleading trends due to the irregular ablation behavior of biotissue because high signals caused by high ablation rates are falsely interpreted as enrichment of elements. Fossilization provides the opportunity to correct such phenomena by standardization with Si as an internal standard. The method demonstrated here allows for quantitative image acquisition without time-consuming sample preparation steps by using comparatively safe chemicals. The diversity of tested samples suggests that this sample preparation method is well-suited to achieve reproducible and quantitative element maps of various plant samples.

## Introduction

Inductively coupled plasma mass spectrometry in combination with laser ablation sample introduction (LA-ICPMS) is established as a powerful technique for the analysis of solid samples in various fields.^1,2^ With a spatial resolution in the single digit μm range and low limits of detection (below mg kg^–1^), the method has become an attractive approach for element mapping.^3−5^ With the introduction of fast-washout ablation cells and the combination with the simultaneous multielement detection capabilities of time-of-flight mass spectrometers (TOFMS), multielement analysis with a high throughput has been realized and is currently applied in geology, material sciences, and biology.^4,6−8^ The acquired element distributions can then be interpreted by experts in the respective fields to determine a wide range of information from the samples.^9^ However, a large number of these applications shown are qualitative and show only relative distributions, which indicates that quantification of the acquired signals remains difficult.^9,10^ As LA-ICPMS requires matrix-matched standards for optimal quantification, the analysis of heterogeneous samples remains challenging.^11,12^ The lack of matrix-matched certified reference materials (CRMs) for a variety of biological samples severely limits the quantification capabilities of the method.^11−14^ Furthermore, the introduction or determination of an internal standard often affects samples themselves and results in the loss or alteration of information.^9^ This problem is less pronounced in geological applications, where mineral phases can be distinguished, and corresponding internal standards can be selected. Furthermore, 100 wt % normalization can be applied for quantification which removes the necessity of an internal standard.^15^ However, the complexity of biological samples and dissimilar absorption properties of each molecule result in individual laser pulses experiencing different matrix compositions.^10^ This is increasingly prominent at higher spatial resolution as individual cells have inherently heterogeneous structures.^16^ There have been various approaches for quantification of biomatrices to reduce these effects. The development of organic polymers and gels, which can be reproducibly doped with known concentrations of various elements, can function as external standards and simulate the role of CRMs.^11,17^ Thin sections with known concentrations in combination with droplets for external calibration have also been applied for the quantification of heavy metals within animal tissue samples.^18,19^ Unfortunately, these methods cannot correct for matrix effects and different ablation rates that result from the ablation of heterogeneous and heteromorphous samples. An internal standard is required to correct for unpredictable and unknown ablation rates.^20^ The use of carbon as internal standard has been disputed in the literature.^21^ The ablation behavior depends strongly on the molecular structure combined with formation of gas species makes carbon an unreliable internal standard despite its apparent homogeneity in biotissue.^22,23^ Furthermore, while TOFMS instruments are desired due to their multielement analysis capabilities, modern instruments are often unable to measure carbon without significant sacrifices of sensitivity in higher mass regions.^24^

An alternative to internal standardization to correct varying ablation rates is the use of total-consumption approaches of thin sections.^25^ However, measurement durations increase by orders of magnitude if more than one laser pulse is required, and any variations in thickness between the standard and sample will lead to errors. Furthermore, the production of thin sections is not possible for every type of sample and involves the risk of adding impurities or altering the three-dimensional structure.^26^ Alteration of the three-dimensional structure is also observed if samples are dried prior to the measurements, as cells collapse upon the removal of water. Direct analysis of hydrated samples results in vaporization of the water of the cells in a sort of steam explosion which can damage the structure, resulting in irregular ablation behavior and changes the excitation conditions within the ICP.^27^

This work proposes the use of in vitro fossilization of plant-tissue to address some of the challenges listed above by replacing water within the complex structure of fresh biological samples by a silicate matrix within 24 h.^28^ This solidification of the complex matrix with a mineral-like phase stabilizes the three-dimensional structure of the sample during the measurement and provides improved reproducibility of the ablation process. As an added benefit, silicon can be utilized as an internal standard to correct for different ablation rates of the standard and sample. The addition of a silicate phase makes the use of the common NIST SRM 610/612 glasses a feasible external standard.^29^ This approach is used for a multielement quantitative mapping of plant-tissues with high spatial resolution using LA-ICP-TOFMS. As proof of concept, this is showcased based on the examples of sunflower (*Helianthus annuus*), soy bean (*Glycine max*), and corn (*Zea mays*). Replicates of the plants grown on nonspiked and on Cd-spiked agricultural soil are compared in order to determine the localization of Ca, Cd, Cu, and Zn uptake within the leaves of common crops.^30,31^

## Experimental Section

### Instrumentation

All measurements were carried out using an ArF excimer laser (193 nm, GeoLas C, Lambda Physik, Göttingen) equipped with a parallel flow ablation cell and using the imaging control system as described by Neff et al.^32^ This allowed for synchronized control of the 3D XYZ stage, laser trigger, and data acquisition, making the acquisition of binned data possible for each laser pulse, which facilitates data evaluation. The ablation cell was flushed with a mixture of helium (1.5–1.6 L min^–1^) and argon (0.8–0.9 L min^–1^). The ablation cell was coupled to an ICP-TOFMS instrument (icpTOF2R, TOFWERK AG, Thun, Switzerland) for quasi-simultaneous detection of the mass range of *m*/*z* = 14–254.^33^ The instrument was tuned for high sensitivity, low oxide rates (^232^Th^16^O/^232^Th < 1%), and a ^238^U/^232^Th ratio of 1–1.1 for LA-ICP-TOFMS analysis of NIST SRM 610.

Measurements of liquid samples of digested plant material were carried out using radial ICP-OES (Spectro Arcos, Spectro Analytical Instruments, Germany). The instrument was tuned to maximum sensitivity. It was operated with Ar nebulizer gas (1 L min^–1^) and a pump rotation speed of 30 rpm.

### Sample Treatment and Sample Preparation

Two replicates of sunflower, corn, and soybean plants were grown either in nonspiked or Cd-spiked agricultural soil (added 3 mg kg^–1^ of Cd to 1 kg of soil) each in a greenhouse under controlled conditions. The leaves of the plants were harvested when they were fully expanded, green, and transpiring. Small sections were cut out (approximately 1 cm^2^) from regions of interest, and the samples were divided into two groups, dried, and fossilized. Dried samples were dehydrated for 48 h at 40 °C. For the fossilization, samples were placed in 50 mL PVC tubes without prior treatment or flattening. The fossilization procedure was carried out according to Drum^28^ as follows: 25 mL of ultrapure water (>18.2 MΩ) was added to the tube. A total of 0.1 mL of concentrated hydrochloric acid (p.a. > 37%, Sigma-Aldrich, Germany) was added, and the samples were left to acclimate for 5 min. One gram of sodium metasilicate pentahydrate (99%, abcr, Germany) was added to the solution, which results in a pH of 13–14. The tube was closed and vigorously shaken to dissolve the sodium metasilicate before adding a magnetic stir bar and being gently stirred. After 24 h, the fossilized leaf tissue was removed from the solution and washed with ultrapure water. The sample was then left to dry in-between two microscope slides for 48 h with a 0.5 mm spacer between the slides to allow airflow. The fossilization and drying process did not cause any observable morphological changes within the sample, as already shown by Drum.^28^ After drying, the samples were placed onto double-sided tape and attached to a microscope slide. The sodium metasilicate was analyzed before use and minor impurities of Zr and Hf (low mg kg^–1^ range) were found, which need to be considered in the experimental plan. However, these elements were not relevant to this study.

For the determination of the bulk concentration of the elements within the leaves, samples were first dried for 48 h at 40 °C. The dried samples were then milled and homogenized. Approx. 0.2 g of material was weighed before adding 3 mL of water, 4 mL of sub-boiled HNO_3_ (Sigma-Aldrich, Germany), and 1 mL of H_2_O_2_ (35%, Acros Organics, Netherlands). The samples were digested in a turboWAVE microwave-assisted digestion system (MLS GmBH, Leutenkirch, Germany). Afterward, the sample was concentrated down to 0.3 mL at 180 °C before being diluted to 30 mL with ultrapure water.

### LA Measurement Procedure

Element mapping measurements were carried out in hole-drilling mode at 100 Hz,^32^ with 10 repeat pulses at every spot and a spot size of 10 μm diameter. Hole drilling mode was chosen for this study, as elements of interest were close to the limits of detection for single pulse mode, and the spatial resolution of 10 μm was desired to provide maximum spatial information. Hole-drilling allowed for an increase in ablated material while maintaining spatial resolution in the *X* and *Y* directions. A 200 × 100 pixel image was acquired from each sample in corresponding regions of interest. This resulted in the analysis of a 2 mm × 1 mm area. For quantification, a 20 pixel × 20 pixel area was analyzed on NIST SRM 610 before and after the measurements of the leaf samples. Afterward, a gas blank measurement was recorded for the same duration.

## Results and Discussion

### Process Development

In the first experiments, the process of in vitro fossilization was developed. A 4% sodium metasilicate solution was best suited for fossilization of plant-tissue within 24 h. Solutions containing 2–10% were also tested with comparable results, and similar Si incorporation into the leaves was achieved (based on signal intensities during ablation). Twenty-four hours was chosen to guarantee that the fossilization process had finished. Some samples began to sink in the water due to the increase in density after 4–6 h but were kept in the solution for the total of 24 h to allow complete fossilization. The process required fresh samples containing significant amounts of water to allow replacement by the silicate matrix. Providing appropriate humidity during storage or rehydration procedures could potentially extend the time window for the fossilization process to be used. Variations in solution temperature and the effect on the speed of the process have not been studied but could also affect the time for sample preparation. Surprisingly, the fossilized samples also preserved their green color over the course of months at room temperature, while the dried samples browned already during the drying process and showed signs of ongoing degradation in the following weeks. The fossilized samples also do not undergo any morphological changes over the course of several months when stored at room temperature in closed containers under an air atmosphere. This is also observed in natural silicification where no degradation of cell walls is observed.^34^

In addition to the samples shown in this study, this approach was validated on ficus (*Ficus benjamina*/*Ficus elastica*), wheat (*Triticum aestivum*), and noccaea (*Noccaea caerulescens*). However, tobacco leaves (*Nicotiana tabacum*) dissolved when this experiment was repeated, with mostly vein structures breaking apart and the sample losing its structural integrity. Animal tissue was also tested with pork lung tissue. The tissue did not withstand the high pH (13–14) of the fossilization solution and dissolved rapidly, which was expected, as animal tissue is known not to withstand highly alkaline conditions.^35^ However, it is currently unknown why the veins of tobacco leaves dissolved. Their lamina is significantly smaller than the lamina of the other investigated plants, which might have resulted in clogging with silicate and preventing diffusion.^36^ Unfortunately, due to a lack of applications for in vitro fossilization, research into the mechanisms behind it remains sparse. Even for natural silicification, the exact chemical processes remain poorly understood.^37,38^

### Differences in Ablation Behavior

**Figure 1:**
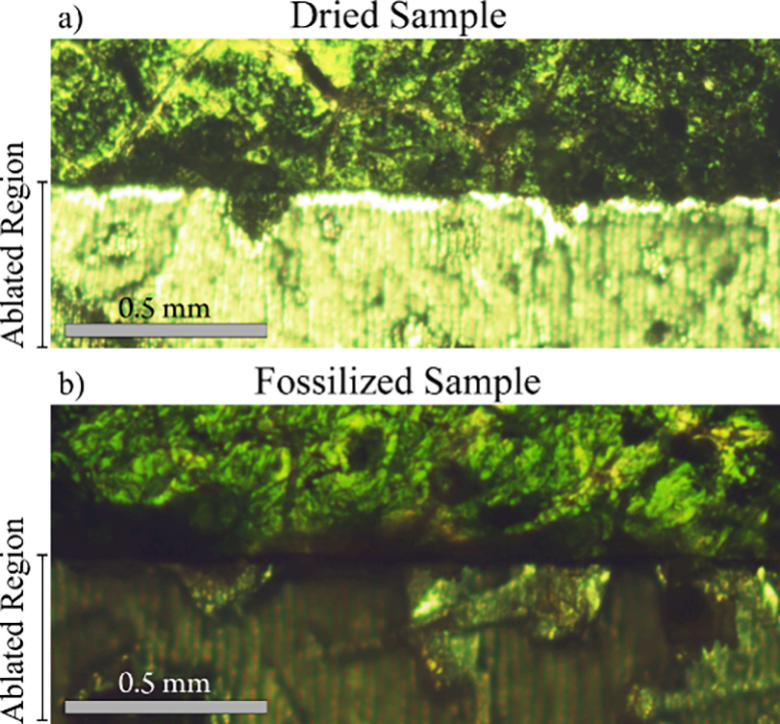
Structural differences due to changes in ablation behavior between dried (a) and fossilized (b) plant-tissue of a sunflower plant. Ablated parts of the leaves are indicated on the *y*-axis. An uneven line with thread-like growths is observed in the dried sample, whereas the fossilized samples show a clean-cut even line at the border of the ablation region.

The ablation behavior of different leaves was then compared between samples that were simply dried and those that were fossilized (Figure 1). It was observed that the dried sample experienced “flaking”, where parts of the sample were removed in large chunks outside the ablation crater. This was most likely caused by the irregular destruction of the cellulose walls during laser ablation. Despite using a significantly higher fluence on the fossilized samples (40 J/cm^2^ vs 15 J/cm^2^), no “flaking” effect was observed. This may be caused by the silicate surrounding the cellulose and maintaining the leaf cell structure. Comparing the edges of the ablated region, the dried samples showcase thread-like structures at the edges. Contrary to the dried samples, ablation on the fossilized samples resulted in a clean-cut edge, as commonly observed during mineral ablation. This ablation behavior resulted in higher spatial resolution and less blurring of the element distribution, which will be discussed further below.

### Element Quantification within Fossilized Tissue

Several fossilized tissue samples of corn were digested and analyzed in preliminary experiments as sufficient amounts of material were available. A consistent Si content of 2 ± 0.3% was observed for all investigated samples, which corresponds to approximately 5 ± 0.8 wt % SiO_2_. With μXRF measurements (M4 Tornado, Bruker Nano GmbH, Berlin, Germany), semiquantitative measurements of other fossilized plants were made in order to determine their degree of silicification relative to the corn samples. This resulted in a ratio of 1:1.5:2 for Si in corn, sunflower, and soybean, respectively. The varying degrees of silicification are assumed to be due to the naturally varying degrees of the water content between these plant species.^39−41^

For quantification, an approach based on 100 wt %-normalization was chosen.^15^ However, a 100 wt %-normalization cannot be directly applied for biological samples as N and O cannot be measured by means of ICP-MS, and C can only be measured on the ICP-TOFMS instrument used when optimizing the instrument for sensitivity in the low mass region, as discussed earlier. Unfortunately, applying such an optimization prevents the detection of the elements of interest (Cd in this case). As such, the quantified image is instead renormalized to a mean SiO_2_ concentration of 5% for corn, 7.5% for sunflowers, and 10% for soy beans. This approach corrects for the elements that could not be analyzed, but make up the majority of the mass.

**Figure 2:**
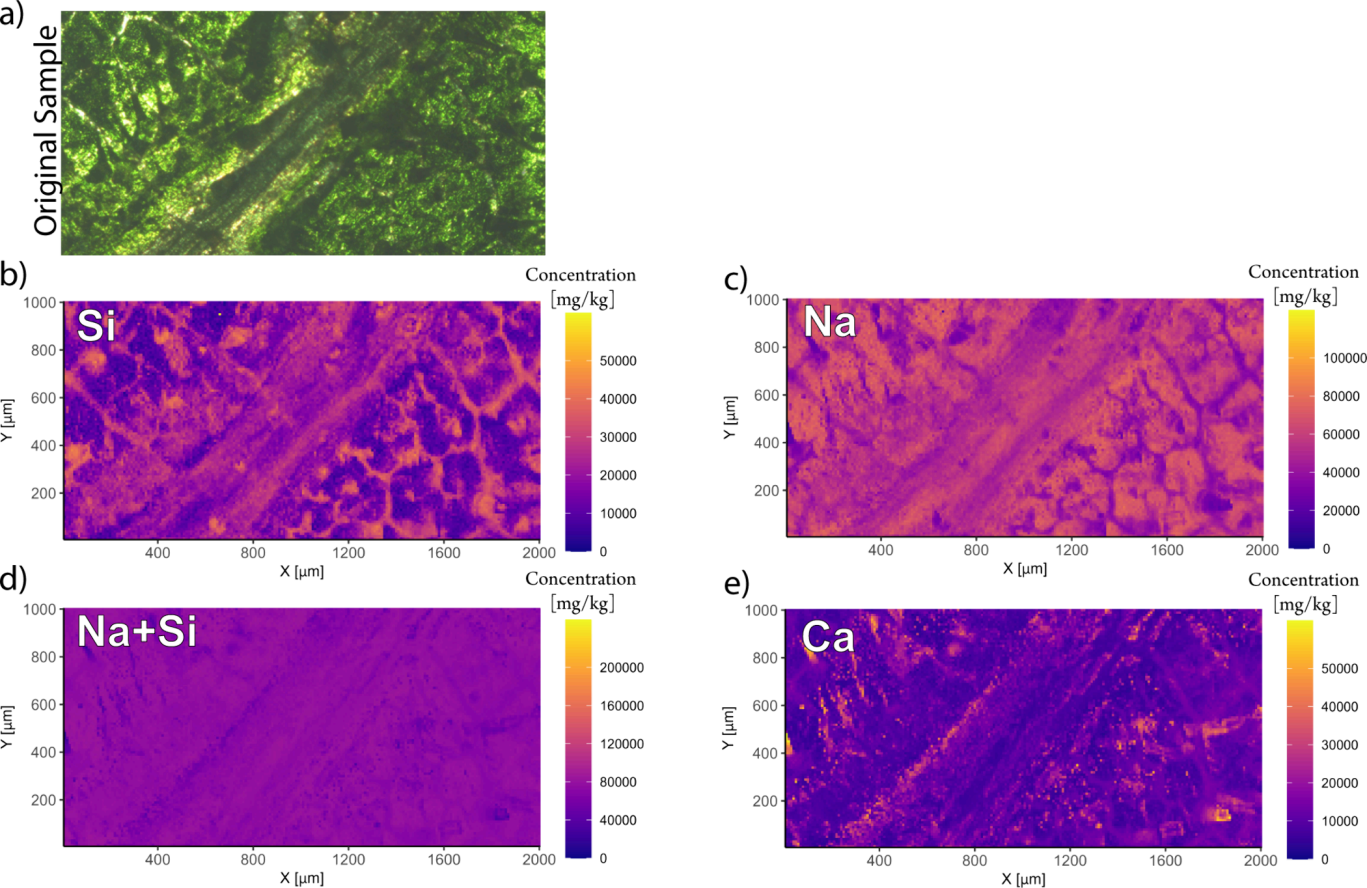
Element distribution after fossilization based on a sunflower leaf. The distribution of the sum of Na and Si is homogeneous with the exception of calcium inclusions in the leaf. (a) Microscope image preablation of the analyzed area, (b) Si element distribution, (c) Na element distribution, (d) combined Na and Si element distribution, and (e) Ca element distribution revealing a negative correlation with the sum of the distributions of Na and Si.

As sodium metasilicate was used for the fossilization, an incorporation of 5–10% Na was measured in addition to the silicate assumed to be in the form of Na_2_CO_3_. In areas where Si was enriched, it correlated with a depletion of Na. The reason for the different inclusion trends are currently unknown and will require further investigation. However, the sums of the concentrations of the two elements remained reproducibly within a relative standard deviation of <8% across the analyzed area for all samples. Exceptions that corresponded to mineral inclusions within the leaf tissue of sunflower were also found (Figure 2). Depletions in the sum of Si and Na correlated with inclusions of Ca (presumably calcite) within the leaf. The Pearson correlation coefficient between the Ca concentration and the combined Na and Si concentrations resulted in −0.96, revealing a significant inverse correlation between those elements. This does not limit the quantification capabilities of the method, as the ablation behavior of mineral inclusions is comparable to the silicate, and such mineral phases can be included in the 100 wt %-normalization. The observed even distribution of silicate, it indicates that a reproducible fossilization process can be achieved. The sum of the distributions of SiO_2_ and Na_2_CO_3_ allows for the correction of varying ablation rates, but their incorporation likewise prevents the quantification of endogenous Na and Si within the tissue.

Sample digestion (see Table S1) and LA-ICP-TOFMS imaging agree within a factor of 2.5 in the quantification for all measured elements. Deviations were expected, as the LA-ICP-TOFMS mapping process was carried out on a small area of a heterogeneous sample at regions of interest, while whole leaves were used to determine the average concentration after digestion. Concentrations of Na and Si within different leaves will vary depending on the different degrees of fossilization of each plant type, which has to be taken into account for the normalization. Furthermore, smaller deviations in the quantification are expected as fossilization marginally changes the density of the tissue, and quantification is made based on the fossilized sample and not on the naturally preserved sample. As fossilization extends the matrix by 10–15 wt % when compared to a fully dehydrated natural sample, minor variations are expected, while the relative ratios of each pixel should not be affected. These deviations could then be corrected to calculate the corresponding concentrations in a dried sample, if preferred. Independent of the stated differences, the majority of the determined concentrations supports the usefulness of such a quantification procedure. Deviations of a factor of 2–3 from leaf to leaf due to biological processes have been published previously which further validate our quantification approach.^42^ Similar differences can be observed between the digested data within this study as well as seen based on the standard deviations (see Table S1). Importantly, the correct degree of silicification must be determined for each plant type. If the respective variations are not accounted for, the concentrations of other elements within the leaves are inaccurately estimated. To prevent this and to make the concentrations between different plant-types comparable for future studies, each species needs to be digested after fossilization to provide plant-specific internal standard concentrations, which can then be applied for imaging. Furthermore, increasing the size of the acquired image would allow a more representative sampling of the leaves, which would provide increased comparability between bulk digestion and the imaging. However, the range of concentrations of the element maps is consistent across multiple adjacent locations on the leaves analyzed on different days, which supports the reproducibility of this approach.

**Figure 3:**
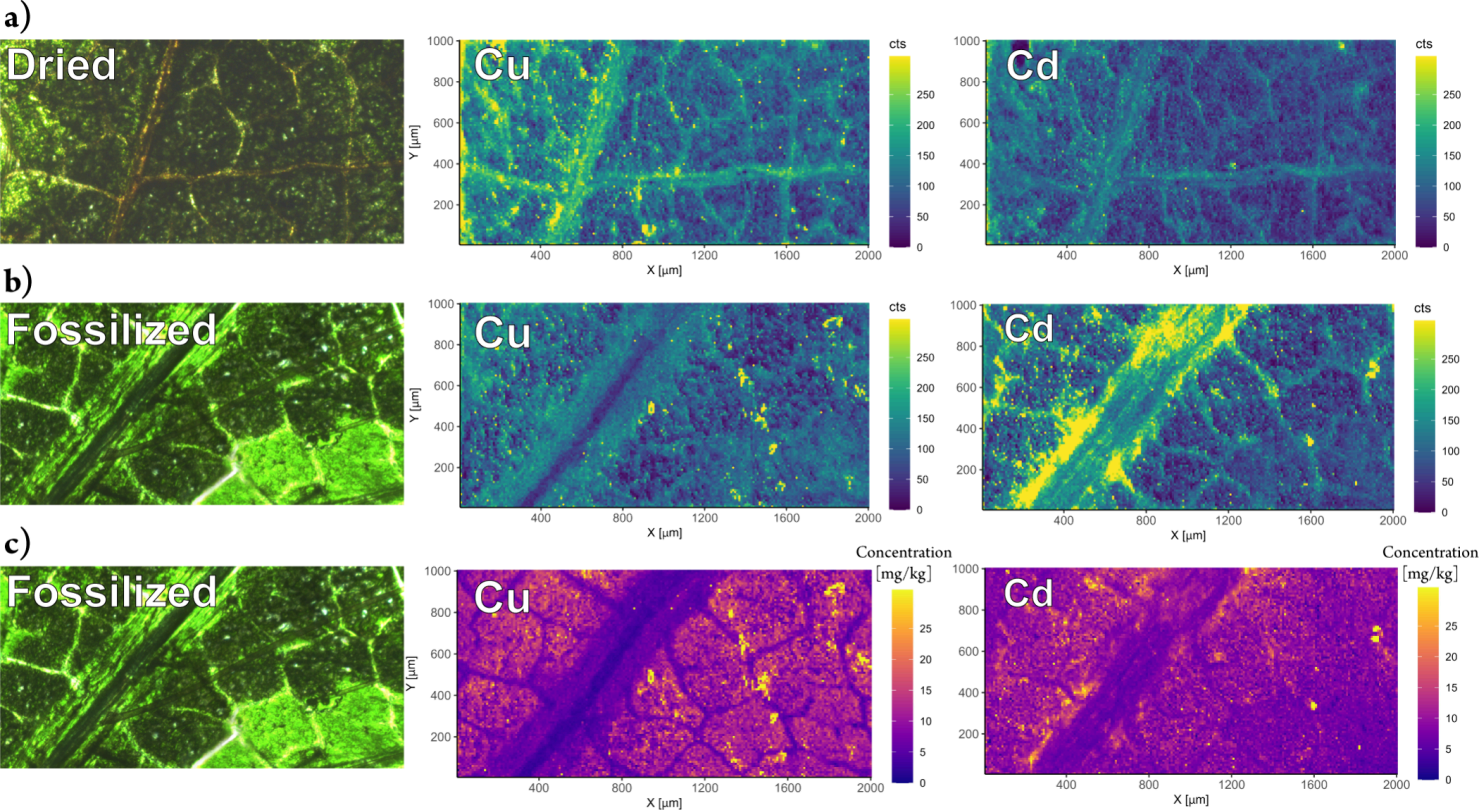
Comparison of dried and fossilized samples of a soybean leaf grown in Cd-spiked agricultural soil. The location was a side vein in the middle section of the leaf. (a) Microscopy image of the dried sample prior to the ablation process, as well as Cu and Cd raw intensity maps. (b) Microscopy image of the fossilized sample prior to the ablation process, as well as Cu and Cd raw intensity maps. (c) Microscopy image of the fossilized sample prior to the ablation process, as well as quantitative Cu and Cd concentration maps of (b). Quantification of (a) is not possible due to lack of internal standard available, whereas Si can be used for (b).

When the acquired element distributions of dried leaf samples are compared with those of fossilized samples, several effects are observed (Figure 3). First, the fossilized sample can be quantified while the dried sample can only be shown as an intensity map, which would not allow for comparison of the heavy metal uptake rates between different plants. Second, the visual sharpness in the fossilized sample is improved compared to the dried sample which is especially noticeable with Cu. This is due to the previously mentioned improved ablation behavior after fossilization and absence of “flaking” during ablation. The Cd content of the plant is near the limit of quantification (approximately 2 ppm, based on Poisson statistics^43^), which results in significant levels of noise being observed. However, it is discernible that a significant level of enrichment takes place next to the vein of the leaf, while the Cd content decreases at larger distances from the vein. The opposite trend is observed for the intensity map, where it appears as if Cu and Cd would enrich within the veins and thereby the vascular tissues of the leaf (Figure 3). These contradicting trends illustrate the necessity for the ablation rate correction. Here, the significant differences in ablation rates are not only due to the complex matrix but also due to the sample morphology as the lamina of the leaf contain large hollow air spaces. As the surface layers of the lamina are comparatively thin, the signals observed are generally lower as less material is available for ablation. Contrary to the lamina, the veins are thicker, which allows for all 10 laser pulses in the hole-drilling approach to ablate significant amounts of material and consequently increase the signal. However, it is evident from the quantified map that despite higher signals, depletion of these elements is observed in the veins of the plant. Naturally, these misleading effects of intensity maps are more pronounced for hole-drilling, line-scans, and total consumption approaches, where the same location is ablated several times. However, ablation rates can vary significantly for the first laser pulse and affect single-pulse approaches but can still be corrected for with the proposed fossilization approach.

There are currently no calibration methods for LA-ICP-TOFMS analysis of biological samples that can use an internal standardization. As such, it is not possible to correct for varying ablation rates without total consumption. However, total consumption significantly increases the duration of the analysis and requires an exact knowledge of the thickness and density of the sample at every location. Therefore, the introduction of an internal standard in the form of Si into the sample makes the fossilization approach a unique method for quantitative imaging of plant leaves and other biological materials.

The fossilization process does raise the question of how much the distribution of elements may be affected during fossilization. We demonstrate that due to the alkaline conditions during the fossilization, transition metals will not be mobilized. This can be explained by the deprotonation of proteins, peptides, and low molecular weight organic ligands, which will further enhance the complexation of metals and thus immobilize them. Furthermore, if metals were delocalized within the tissue or even removed during the process, we would expect to observe concentration gradients. However, this was only observed for Al as it is mobilized under alkaline conditions as an amphoteric hydroxide (Figure S1). Additionally, the Al signal decreased by 2 orders of magnitude within fossilized tissue when compared to dried tissue. Therefore, the proposed method does not allow for quantification of Al distributions within plant tissue. For all other metals reported in this study, however, it can be confidently assumed that the measured element distribution corresponds to the distribution within the original plant tissue.

The Cu concentration was depleted in the veins and enriched between the veins of soybean leaves (Figure 3). In these green, fully expanded leaves, Cu ranged within typical concentrations in dried plants (10 to 12 mg kg^–1^).^44^ The Cu depletion in the veins suggests that Cu was efficiently removed from xylem vessels in the veins and further distributed to mesophyll cells where it fulfills its biological functions.^45^ In contrast to Cu, Cd concentrations were highest along the major veins, while Cd was depleted in the mesophyll cells. As Cd concentrations reached toxic concentrations above 10 mg kg^–1^, Cd was likely immobilized through detoxification in, e.g., adjacent cells of the xylem vessels before it could be transported to mesophyll cells.^45,46^

**Figure 4:**
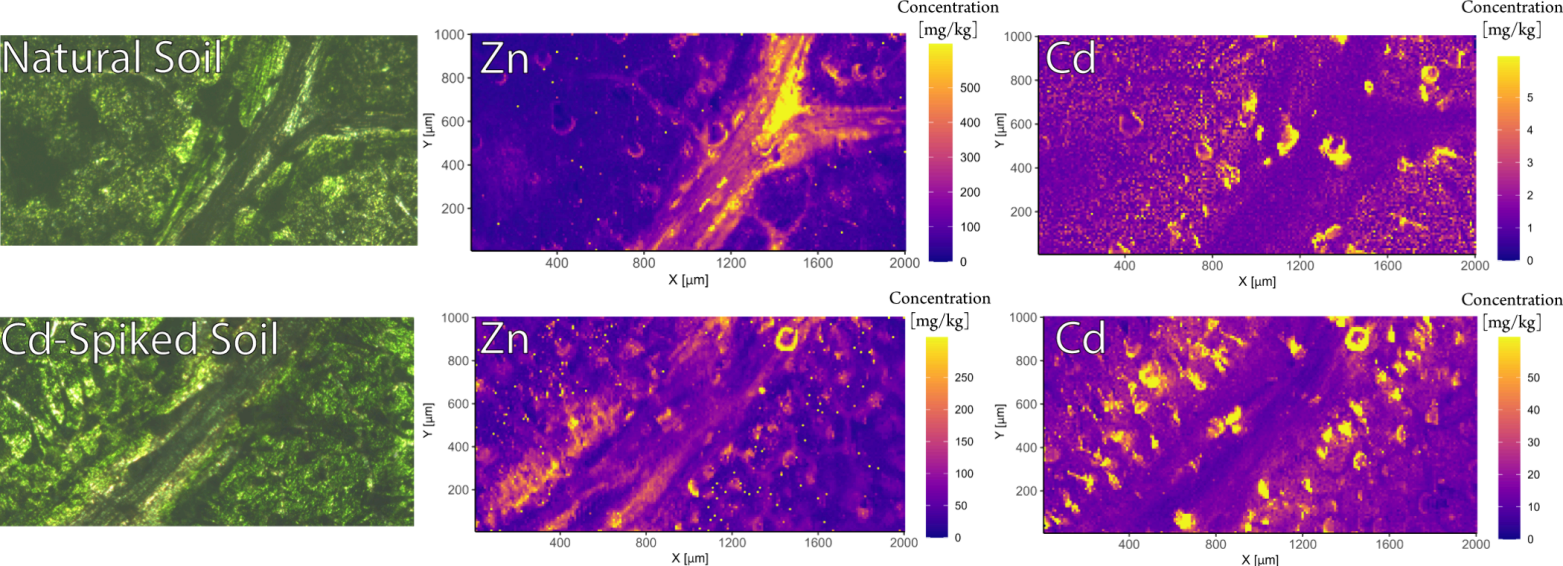
Effect on Cd and Zn distributions within leaves of sunflowers grown in non-spiked agricultural soil vs Cd-spiked agricultural soil (3 mg kg^–1^). Enrichment can be seen at the trichomes (i.e., leaf hairs) for both Zn and Cd. Zn appears to be more depleted in Cd-spiked soil within the vein, while enriched in non-spiked soil. The enrichment of Cd decreases the farther away the trichomes are from the vein. Note the different scales of each picture.

The distribution of Zn in sunflower leaves strongly differed when Cd was added to the soil (Figure 4). Without Cd, Zn concentrations reached concentrations of more than 500 mg kg^–1^ in the major vein. Such a high Zn concentration can have toxic effects, based on tests conducted with bulk concentrations.^47^ Our experiments indicate that sunflowers can withstand a significantly higher concentration than that proposed based on bulk concentrations. With Cd addition to the soil, Zn concentrations decreased which aligns with well-studied Zn and Cd competition effects in plants.^48,49^ Finally, the accumulation of Cd and Zn in the trichomes confirms (nonquantified) elemental maps of plant leaves that were acquired with synchrotron X-ray techniques.^50−52^ The LA-ICP-TOFMS maps in the fossilized samples obtained here additionally showed that Cd concentrations in trichomes reached >50 mg kg^–1^. As the bulk leaf concentration was similar compared to the Cd concentrations in the trichomes (Table S1), these results suggest that Cd accumulation in the trichomes is the major strategy of sunflowers to cope with high Cd levels in leaves.

## Conclusion

In vitro fossilization is a valuable sample preparation method for spatially resolved multielement quantification of plant-tissue by means of LA-ICP-TOFMS. The addition of SiO_2_ and Na_2_CO_3_ into the sample matrix results in a mineral-like ablation behavior, which improves the spatial resolution by reducing flaking behavior. Furthermore, the mostly homogeneous fossilization process allows for the use of 100 wt %-normalization, followed by an internal standardization of the sample and thus correction of different ablation rates. Also, silicate reference materials can be used for quantification. Data corrected for ablation rate show that apparent trends in uncorrected signal maps may provide misleading information for which previous approaches could not correct for. The quantitative results of our proof-of-concept study indicate that element concentrations similar to those of bulk data can be achieved. Plant specific determination of the rate of fossilization must be considered and will contribute to the accuracy of the results. However, some limitations remain with the fossilization approach presented in this study and will require further detailed investigation. First, the use of sodium metasilicate to incorporate a silicate structure into the leaves prevents the quantification of endogenous Na and Si within the tissue. This can be circumvented by using a different approach for the fossilization based on, for example, calcites, as observed in natural fossilization. Second, this approach results in a strongly basic solution (pH 13–14) in which the tissue sample must remain stable for approximately 24 h as was the case here for five out of six plant species. The mobilization of endogenous ions is unlikely at high pH and was observed only for Al in this study. Plant tissue was observed to be stable in the majority of cases; however, animal tissue did not withstand such alkaline environments.

Nevertheless, most of these challenges might be solved by varying the chemicals used for implementing an internal standard into the biological matrix. In vitro fossilization has been described at different pH levels even for animal tissue by using different variations of silicic acid.^53^ Further process optimization could also improve the degree of fossilization, the duration of the process, and the size of the samples that can be analyzed and allow for more widespread application with all types of tissues. However, the possibility presented here to do high-throughput, multielemental, quantitative analysis of plant-tissue at high spatial resolutions opens the doors to improve the understanding of how plants deal with deficiencies and toxicities caused by nutrients and pollutants.

## Supporting Information Available

Concentrations determined by means of bulk digestion measurements of samples grown on spiked and nonspiked agricultural soil (Table S1); Al distribution within a soybean leaf showing concentration gradient caused by washout in the alkaline solution (Figure S1); various element distributions in all the analyzed samples shown within this study (Figures S2–S7) (PDF)

## References

[ref1] Van Acker T.; Van Malderen S. J. M.; Van Helden T.; Stremtan C.; Šala M.; Van Elteren J. T.; Vanhaecke F. Analytical figures of merit of a low-dispersion aerosol transport system for high-throughput LA-ICP-MS analysis. J. Anal. At. Spectrom. 2021, 36, 1201–1209. 10.1039/D1JA00110H.

[ref2] Günther D.; Hattendorf B. Solid sample analysis using laser ablation inductively coupled plasma mass spectrometry. Trends Anal. Chem. 2005, 24, 255–265. 10.1016/j.trac.2004.11.017.

[ref3] Wang S.; Brown R.; Gray D. J. Application of laser ablation-ICPMS to the spatially resolved micro-analysis of biological tissue. Appl. Spectrosc. 1994, 48, 1321–1325. 10.1366/0003702944028001.

[ref4] Kindness A.; Sekaran C. N.; Feldmann J. Two-Dimensional Mapping of Copper and Zinc in Liver Sections by Laser Ablation - Inductively Coupled Plasma Mass Spectrometry. Clin. Chem. 2003, 49, 1916–1923. 10.1373/clinchem.2003.022046. 14578324

[ref5] Van Malderen S. J. M.; Van Acker T.; Vanhaecke F. Sub-micrometer Nanosecond LA-ICP-MS Imaging at Pixel Acquisition Rates above 250 Hz via a Low-Dispersion Setup. Anal. Chem. 2020, 92, 5756–5764. 10.1021/acs.analchem.9b05056. 32202412

[ref6] Wang H. A. O.; Grolimund D.; Giesen C.; Borca C. N.; Shaw-Stewart J. R. H.; Bodenmiller B.; Günther D. Fast chemical imaging at high spatial resolution by laser ablation inductively coupled plasma mass spectrometry. Anal. Chem. 2013, 85, 10107–10116. 10.1021/ac400996x. 23957530

[ref7] Gundlach-Graham A.; Günther D. Toward faster and higher resolution LA-ICPMS imaging: On the co-evolution of la cell design and ICPMS instrumentation. Anal. Bioanal. Chem. 2016, 408, 2687–2695. 10.1007/s00216-015-9251-8. 26753979

[ref8] Van Malderen S. J. M.; Managh A. J.; Sharp B. L.; Vanhaecke F. Recent developments in the design of rapid response cells for laser ablation-inductively coupled plasma-mass spectrometry and their impact on bioimaging applications. J. Anal. At. Spectrom. 2016, 31, 423–439. 10.1039/C5JA00430F.

[ref9] Doble P. A.; de Vega R. G.; Bishop D. P.; Hare D. J.; Clases D. Laser ablation-inductively coupled plasma-mass spectrometry imaging in biology. Chem. Rev. 2021, 121, 11769–11822. 10.1021/acs.chemrev.0c01219. 34019411

[ref10] New E. J.; Wimmer V. C.; Hare D. J. Promises and Pitfalls of Metal Imaging in Biology. Cell Chem. Biol. 2018, 25, 7–18. 10.1016/j.chembiol.2017.10.006. 29153850

[ref11] Metarapi D.; Schweikert A.; Jerse A.; Schaier M.; Van Elteren J. T.; Koellensperger G.; Theiner S.; Sala M. Semiquantitative Analysis for High-Speed Mapping Applications of Biological Samples Using LA-ICP-TOFMS. Anal. Chem. 2023, 95, 7804–7812. 10.1021/acs.analchem.3c01439. 37122168 PMC10193359

[ref12] Schweikert A.; Theiner S.; Wernitznig D.; Schoeberl A.; Schaier M.; Neumayer S.; Keppler B. K.; Koellensperger G. Micro-droplet-based calibration for quantitative elemental bioimaging by LA-ICPMS. Anal. Bioanal. Chem. 2022, 414, 485–495. 10.1007/s00216-021-03357-w. 33954828 PMC8748332

[ref13] Durrant S. F.; Ward N. I. Recent biological and environmental applications of laser ablation inductively coupled plasma mass spectrometry (LA-ICP-MS). J. Anal. At. Spectrom. 2005, 20, 821–829. 10.1039/b502206a.

[ref14] Hare D.; Austin C.; Doble P. Quantification strategies for elemental imaging of biological samples using laser ablation-inductively coupled plasma-mass spectrometry. Analyst 2012, 137, 1527–1537. 10.1039/c2an15792f. 22314636

[ref15] Liu Y.; Hu Z.; Gao S.; Günther D.; Xu J.; Gao C.; Chen H. In situ analysis of major and trace elements of anhydrous minerals by LA-ICP-MS without applying an internal standard. Chem. Geol. 2008, 257, 34–43. 10.1016/j.chemgeo.2008.08.004.

[ref16] Vogel A.; Venugopalan V. , Mechanisms of pulsed laser ablation of biological tissues. Chem. Rev. 2003, 103, 577–644. 10.1021/cr010379n. 12580643

[ref17] Limbeck A.; Galler P.; Bonta M.; Bauer G.; Nischkauer W.; Vanhaecke F. Recent advances in quantitative LA-ICP-MS analysis: Challenges and solutions in the life sciences and environmental chemistry. Anal. Bioanal. Chem. 2015, 407, 6593–6617. 10.1007/s00216-015-8858-0. 26168964 PMC4545187

[ref18] Schoeberl A.; Gutmann M.; Theiner S.; Schaier M.; Schweikert A.; Berger W.; Koellensperger G. Cisplatin Uptake in Macrophage Subtypes at the Single-Cell Level by LA-ICP-TOFMS Imaging. Anal. Chem. 2021, 93, 16456–16465. 10.1021/acs.analchem.1c03442. 34846133 PMC8674877

[ref19] Van Acker T.; Buckle T.; Van Malderen S. J. M.; van Willigen D. M.; van Unen V.; van Leeuwen F. W. B.; Vanhaecke F. High-resolution imaging and single-cell analysis via laser ablation-inductively coupled plasma-mass spectrometry for the determination of membranous receptor expression levels in breast cancer cell lines using receptor-specific hybrid tracers. Anal. Chim. Acta 2019, 1074, 43–53. 10.1016/j.aca.2019.04.064. 31159938

[ref20] Longerich H. P.; Jackson S. E.; Günther D. Laser ablation inductively coupled plasma mass spectrometric transient signal data acquisition and analyte concentration calculation. J. Anal. At. Spectrom. 1996, 11, 899–904. 10.1039/JA9961100899.

[ref21] Todoli J. L.; Mermet J. M. Study of polymer ablation products obtained by ultraviolet laser ablation - inductively coupled plasma atomic emission spectrometry. Spectrochim. acta, Part B At. Spectrosc., 1998, 53, 1645–1656. 10.1016/S0584-8547(98)00219-5.

[ref22] Frick D. A.; Günther D. Fundamental studies on the ablation behaviour of carbon in LA-ICP-MS with respect to the suitability as internal standard. J. Anal. At. Spectrom. 2012, 27, 1294–1303. 10.1039/c2ja30072a.

[ref23] Van Helden T.; Mervič K.; Nemet I.; van Elteren J. T.; Vanhaecke F.; Rončević S.; Šala M.; Van Acker T. Evaluation of two-phase sample transport upon ablation of gelatin as a proxy for soft biological matrices using nanosecond laser ablation – inductively coupled plasma – mass spectrometry. Anal. Chim. Acta 2024, 1287, 342089 10.1016/j.aca.2023.342089. 38182382

[ref24] Borovinskaya O.; Hattendorf B.; Tanner M.; Gschwind S.; Günther D. A prototype of a new inductively coupled plasma time-of-flight mass spectrometer providing temporally resolved, multi-element detection of short signals generated by single particles and droplets. J. Anal. At. Spectrom. 2013, 28, 226–233. 10.1039/C2JA30227F.

[ref25] O’Reilly J.; Douglas D.; Braybrook J.; So P. W.; Vergucht E.; Garrevoet J.; Vekemans B.; Vincze L.; Goenaga-Infante H. A novel calibration strategy for the quantitative imaging of iron in biological tissues by LA-ICP-MS using matrix-matched standards and internal standardisation. J. Anal. At. Spectrom. 2014, 29, 1378–1384. 10.1039/C4JA00002A.

[ref26] Bouchet-Marquis C.; Hoenger A. Cryo-electron tomography on vitrified sections: A critical analysis of benefits and limitations for structural cell biology. Micron 2011, 42, 152–162. 10.1016/j.micron.2010.07.003. 20675145

[ref27] Wang Y.; Wei X.; Liu J. H.; Wu C. X.; Zhang X.; Chen M. L.; Wang J. H. Cryogenic Laser Ablation in a Rapid Cooling Chamber Ensures Excellent Elemental Imaging in Fresh Biological Tissues. Anal. Chem. 2022, 94, 8547–8553. 10.1021/acs.analchem.2c01736. 35653437

[ref28] Drum R. W. Petrifaction of Plant Tissue in the Laboratory. Nature 1968, 218, 784–785. 10.1038/218784b0.

[ref29] Jochum K. P.; Weis U.; Stoll B.; Kuzmin D.; Yang Q.; Raczek I.; Jacob D. E.; Stracke A.; Birbaum K.; Frick D. A.; Günther D.; Enzweiler J. Determination of reference values for NIST SRM 610–617 glasses following ISO guidelines. Geostand. Geoanalytical Res. 2011, 35, 397–429. 10.1111/j.1751-908X.2011.00120.x.

[ref30] January M. C.; Cutright T. J.; Van Keulen H.; Wei R. Hydroponic phytoremediation of Cd, Cr, Ni, As, and Fe: Can Helianthus annuus hyperaccumulate multiple heavy metals?. Chemosphere 2008, 70, 531–537. 10.1016/j.chemosphere.2007.06.066. 17697697

[ref31] Kötschau A.; Büchel G.; Einax J. W.; Fischer C.; von Tümpling W.; Merten D. Mapping of macro and micro elements in the leaves of sunflower (Helianthus annuus) by Laser Ablation-ICP-MS. Microchem. J. 2013, 110, 783–789. 10.1016/j.microc.2012.12.011.

[ref32] Neff C.; Keresztes Schmidt P.; Garofalo P. S.; Schwarz G.; Günther D. Capabilities of automated LA-ICP-TOFMS imaging of geological samples. J. Anal. At. Spectrom. 2020, 35, 2255–2266. 10.1039/D0JA00238K.

[ref33] Burger M.; Schwarz G.; Gundlach-Graham A.; Käser D.; Hattendorf B.; Günther D. Capabilities of laser ablation inductively coupled plasma time-of-flight mass spectrometry. J. Anal. At. Spectrom. 2017, 32, 1946–1959. 10.1039/C7JA00236J.

[ref34] Dietrich D.; Lampke T.; Rößler R. A microstructure study on silicified wood from the Permian Petrified Forest of Chemnitz. Palaontologische Zeitschrift 2013, 87, 397–407. 10.1007/s12542-012-0162-0.

[ref35] Howell J. M. Alkaline ingestions. Ann. Emerg. Med. 1986, 15, 820–825. 10.1016/S0196-0644(86)80382-1. 3524323

[ref36] Ganeriwala S. N. Relationship between morphology and mechanical properties of tobacco leaf lamina. J. Mater. Sci. 1990, 25, 2199–2206. 10.1007/BF01045790.

[ref37] Huestis L. Chemistry of fossilization. J. Chem. Educ. 1976, 53, 270–273. 10.1021/ed053p270.

[ref38] Allison P. A.; Bottjer D. J.; Allison P. A.; Bottjer D. J. Taphonomy Process and Bias Through Time, 2011 Springer, vol. 32, DOI: 10.1007/978-90-481-8643-3.

[ref39] Neto A. J. S.; Lopes D. D. C.; da Silva T. G. F.; Ferreira S. O.; Grossi J. A. S. Estimation of leaf water content in sunflower under drought conditions by means of spectral reflectance. Eng. Agric. Environ. Food 2017, 10, 104–108. 10.1016/j.eaef.2016.11.006.

[ref40] Hossain M. M.; Liu X.; Qi X.; Lam H. M.; Zhang J. Differences between soybean genotypes in physiological response to sequential soil drying and rewetting. Crop J. 2014, 2, 366–380. 10.1016/j.cj.2014.08.001.

[ref41] Crusiol L. G. T.; Sun L.; Sun Z.; Chen R.; Wu Y.; Ma J.; Song C. In-Season Monitoring of Maize Leaf Water Content Using Ground-Based and UAV-Based Hyperspectral Data. Sustainability 2022, 14, 1–19. 10.3390/su14159039.

[ref42] Hanway J. J.; Dunphy E. J.; Loberg G. L.; Shibles R. M. Dry Weights and Chemical Composition of Soybean Plant Parts Throughout the Growing Season. J. Plant Nutr. 1984, 7, 1453–1475. 10.1080/01904168409363294.

[ref43] Currie L. A. Limits for Qualitative Detection and Quantitative Determination: Application to Radiochemistry. Anal. Chem. 1968, 40, 586–593. 10.1021/ac60259a007.

[ref44] Wiggenhauser M.; Moore R. E. T.; Wang P.; Bienert G. P.; Laursen K. H.; Blotevogel S. Stable Isotope Fractionation of Metals and Metalloids in Plants*:* A Review. Front. Plant Sci. 2022, 13, 1–26. 10.3389/fpls.2022.840941. PMC9063737 35519812

[ref45] Olsen L. I.; Palmgren M. G. Many rivers to cross: The journey of zinc from soil to seed. Front. Plant Sci. 2014, 5, 1–6. 10.3389/fpls.2014.00030. PMC3921580 24575104

[ref46] White P. J. Heavy metal toxicity in plants. Plant Stress Physiol. 2012, 210–237. 10.1079/9781845939953.0210.

[ref47] Broadley M. R.; White P. J.; Hammond J. P.; Zelko I.; Lux A. Zinc in plants. New Phytol. 2007, 173, 677–702. 10.1111/j.1469-8137.2007.01996.x. 17286818

[ref48] McLaughlin M. J.; Smolders E.; Zhao F. J.; Grant C.; Montalvo D. Managing cadmium in agricultural systems; Elsevier Inc., 1st edn., 2021, Vol. 166.

[ref49] Maret W.; Moulis J.-M. Cadmium: From toxicity to essentiality, 2013 Springer, vol. 11.

[ref50] Isaure M. P.; Huguet S.; Meyer C. L.; Castillo-Michel H.; Testemale D.; Vantelon D.; Saumitou-Laprade P.; Verbruggen N.; Sarret G. Evidence of various mechanisms of Cd sequestration in the hyperaccumulator Arabidopsis halleri, the non-accumulator Arabidopsis lyrata, and their progenies by combined synchrotron-based techniques. J. Exp. Bot. 2015, 66, 3201–3214. 10.1093/jxb/erv131. 25873676

[ref51] Li C.; Wang P.; Van Der Ent A.; Cheng M.; Jiang H.; Read T. L.; Lombi E.; Tang C.; De Jonge M. D.; Menzies N. W.; Kopittke P. M. Absorption of foliar-applied zn in sunflower (helianthus annuus): importance of the cuticle, stomata and trichomes. Ann. Bot. 2019, 123, 57–68. 10.1093/aob/mcy135. 30020418 PMC6344099

[ref52] Ricachenevsky F. K.; Punshon T.; Salt D. E.; Fett J. P.; Guerinot M. L. Arabidopsis thaliana zinc accumulation in leaf trichomes is correlated with zinc concentration in leaves. Sci. Rep. 2021, 11, 1–10. 10.1038/s41598-021-84508-y. 33674630 PMC7935932

[ref53] Townson J. L.; Lin Y. S.; Chou S. S.; Awad Y. H.; Coker E. N.; Brinker C. J.; Kaehr B. Synthetic fossilization of soft biological tissues and their shape-preserving transformation into silica or electron-conductive replicas. Nat. Commun. 2014, 5, 1–8. 10.1038/ncomms6665. PMC4268709 25482611

