# Supplementary material for: In Vitro Fossilization for High Spatial Resolution Quantification of Elements in Plant-Tissue Using LA-ICP-TOFMS
Source: Anal Chem. 2024 Mar 14;96(12):4952–9. doi: 10.1021/acs.analchem.3c05849 (PMC10975018; doi:10.1021/acs.analchem.3c05849)
Supplement: Supplementary file 1 — ac3c05849_si_001.pdf [file ac3c05849_si_001.pdf]

Supporting Information

In-Vitro Fossilization for Improved Spatial Resolution and Quantification of Elements in Plant-Tissue using LA-ICP-TOFMS

Pascal Becker[†], Thomas Nauser[†], Matthias Wiggenhauser[‡], Beat Aeschlimann[†], Emmanuel Frossard[‡], Detlef Günther[†, *]

[†]Laboratory of Inorganic Chemistry, Department of Chemistry and Applied Biosciences, ETH Zurich, Zurich 8093, Switzerland

[‡]Institute of Agricultural Sciences, ETH Zurich, Eschikon 33, CH-8315 Lindau, Switzerland

*Corresponding author: guenther@inorg.chem.ethz.ch – Tel. +41 44 632 46 87

Table of contents

Table S1. Comparison of determined concentrations by means of full leaf digestion followed by ICP-OES and LA-ICPTOFMS of fossilized samples. 3

Figure S1: Al distribution within a soybean leaf with a concentration gradient....................................3
Figure S2. Element distributions found within a corn leaf grown on unspiked soil. 4
Figure S3. Element distributions found within a corn leaf grown on Cd-spiked soil. 5
Figure S4. Element distributions found within a soybean leaf grown on unspiked soil. * Shows the general leaf structure but does not correspond to the measured area. 6
Figure S5. Element distributions found within a soybean leaf grown on Cd-spiked soil. 7
Figure S6. Element distributions found within a sunflower leaf grown on unspiked soil.......................8
Figure S7. Element distributions found within a sunflower leaf grown on Cd-spiked soil. 9

Table S1. Comparison of determined concentrations by means of full leaf digestion followed by ICP-OES and LA-ICPTOFMS of fossilized samples. No standard deviations are given for the laser ablation data as they represent the average of each pixel of a heterogeneous area. LOD of Cd corresponds to 0.8 mg kg-1

**Digestion [mg kg^{-1}]**	**Mg**	**P**	**Ca**	**Mn**	**Cu**	**Zn**	**Cd**
Soy – non-spiked	3770<br>±210	2000<br>±600	15000<br>±3000	370<br>±3	7.1<br>±2.1	160<br>±3	< LOD
Soy – Spiked	5000<br>±1000	3100<br>±1100	19000<br>±8000	310<br>±190	7.1<br>±1.2	190<br>±70	16<br>±3
Sunflower – non-spiked	4800<br>±2300	2160<br>±150	28400<br>±800	820<br>±60	15.9<br>±1.0	150<br>±80	< LOD
Sunflower – Spiked	6000<br>±3000	1950<br>±230	30300<br>±1100	1050<br>±170	15.9<br>±1.8	245<br>±8	42<br>±7
Corn – non-spiked	1900<br>±40	957<br>±15	3700<br>±700	74<br>±15	5.6<br>±0.8	31.0<br>±1.8	< LOD
Corn – Spiked	4300<br>±1100	960<br>±30	4792<br>±16	91<br>±3	7.7<br>±0.2	30<br>±6	1<br>±0.4
**LA [mg kg^{-1}]**							
Soy – non-spiked	3400	700	13200	220	8	70	< LOD
Soy – Spiked Soil	5000	800	20000	140	8	140	8
Sunflower – non-spiked	7950	1350	18000	750	26	195	< LOD
Sunflower – Spiked	5550	1800	18000	750	20	75	21
Corn – non-spiked	4200	560	5800	80	14	47	< LOD
Corn – Spiked	5000	550	7300	60	10	40	3

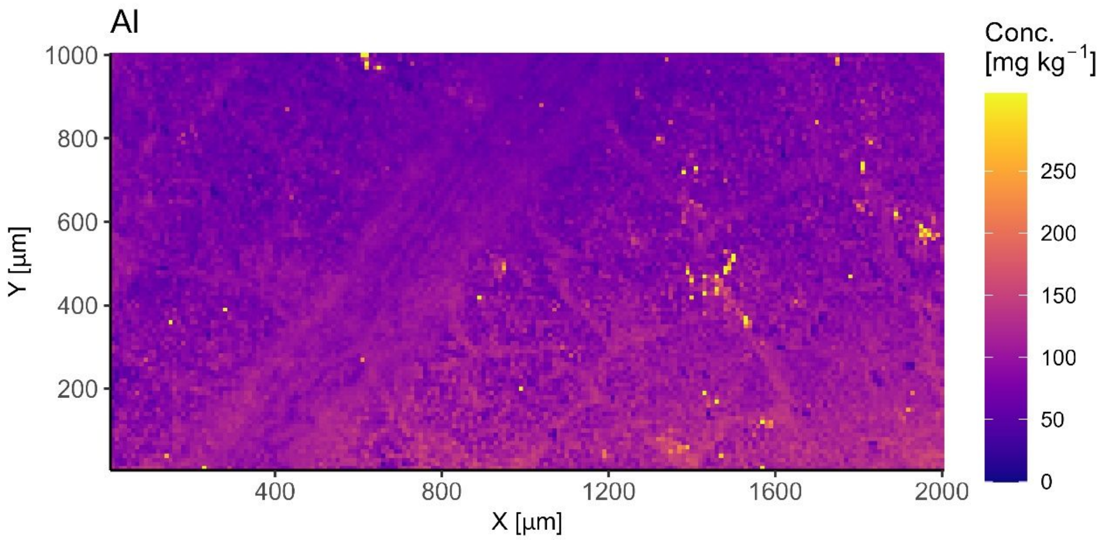

Figure S1: Al distribution within a soybean leaf with a concentration gradient. This is due to the mobilization of Al as a result of the alkaline solution during the fossilization process.

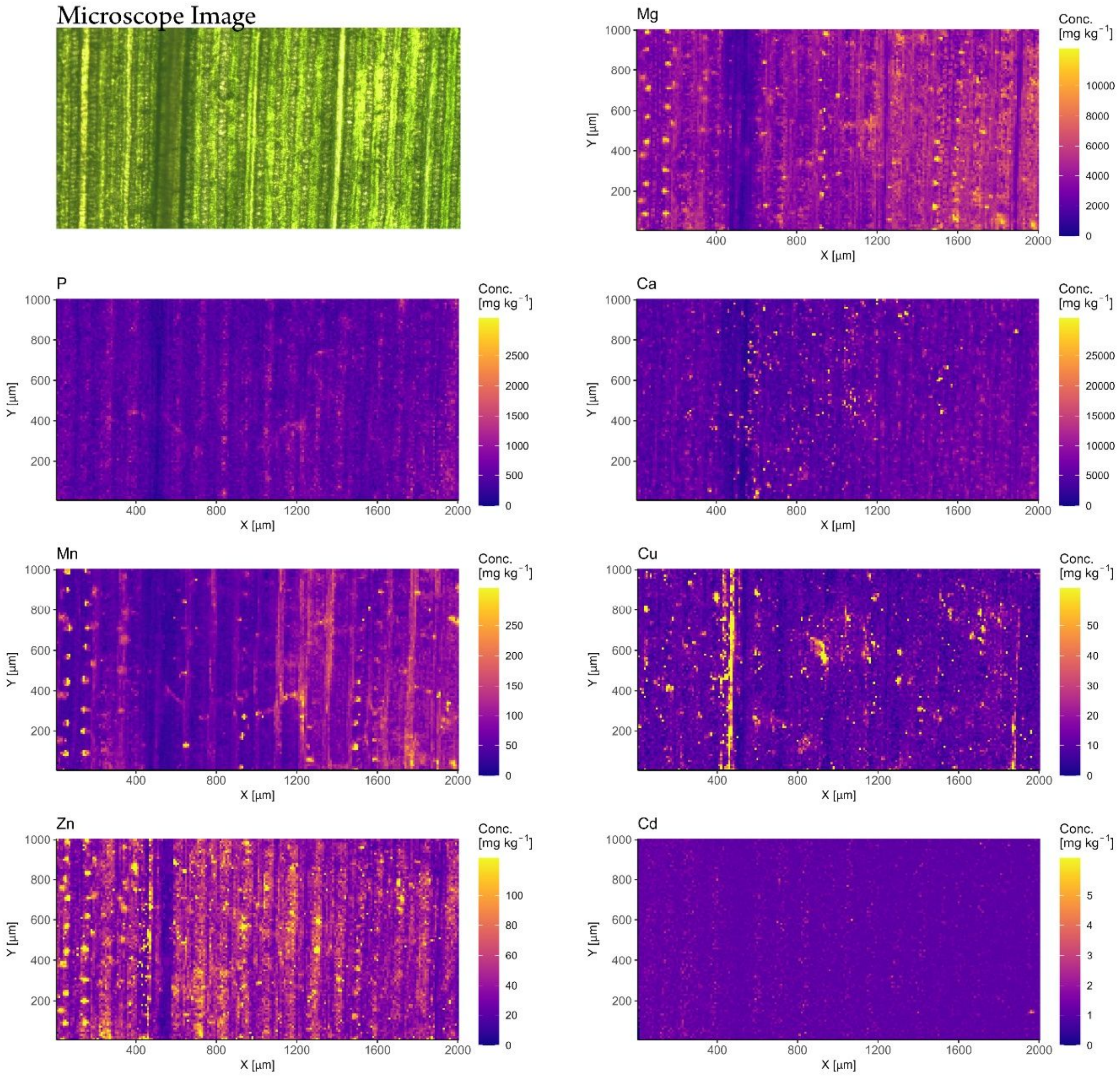

Figure S2. Element distributions found within a corn leaf grown on unspiked soil.

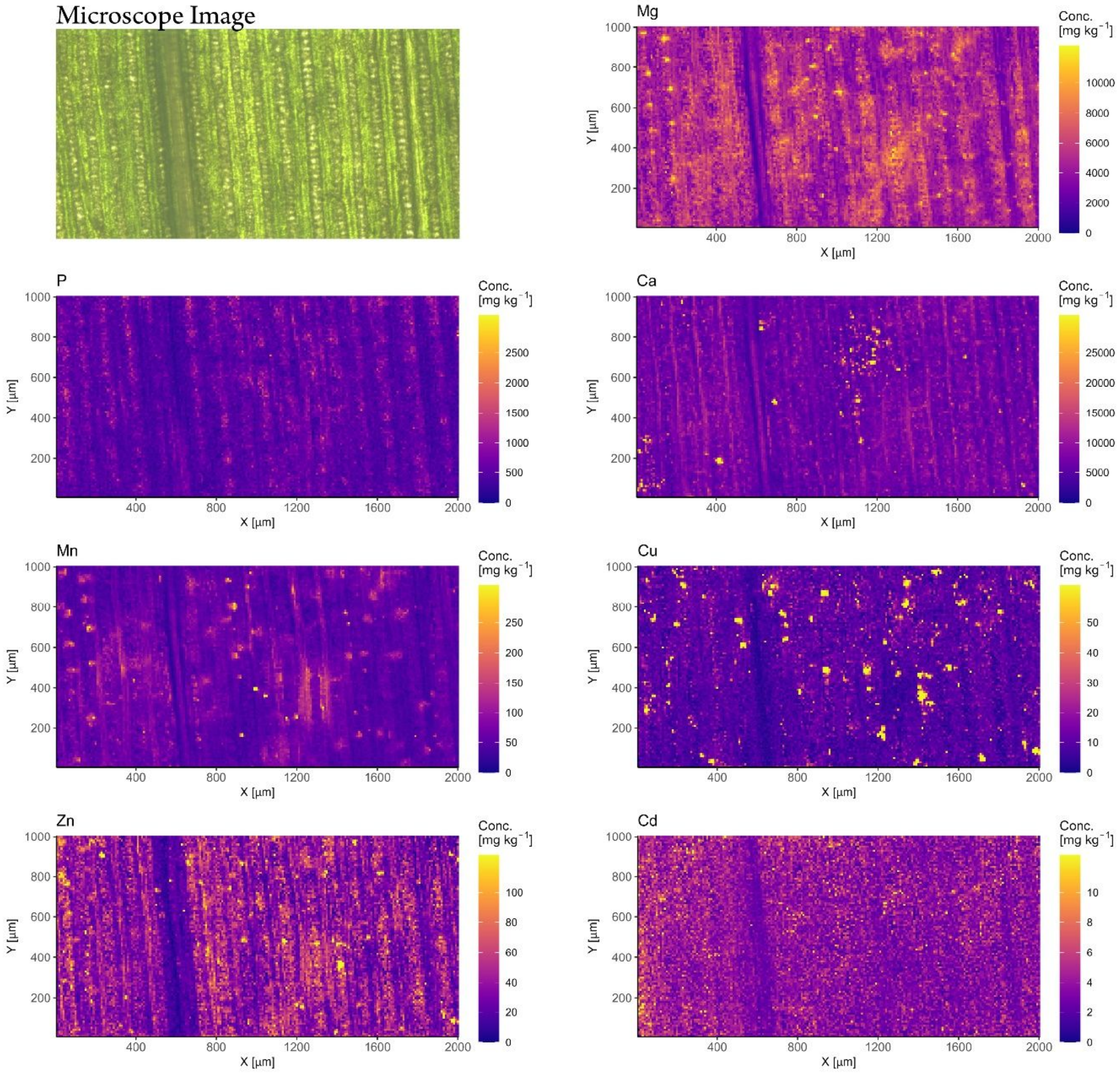

Figure S3. Element distributions found within a corn leaf grown on Cd-spiked soil.

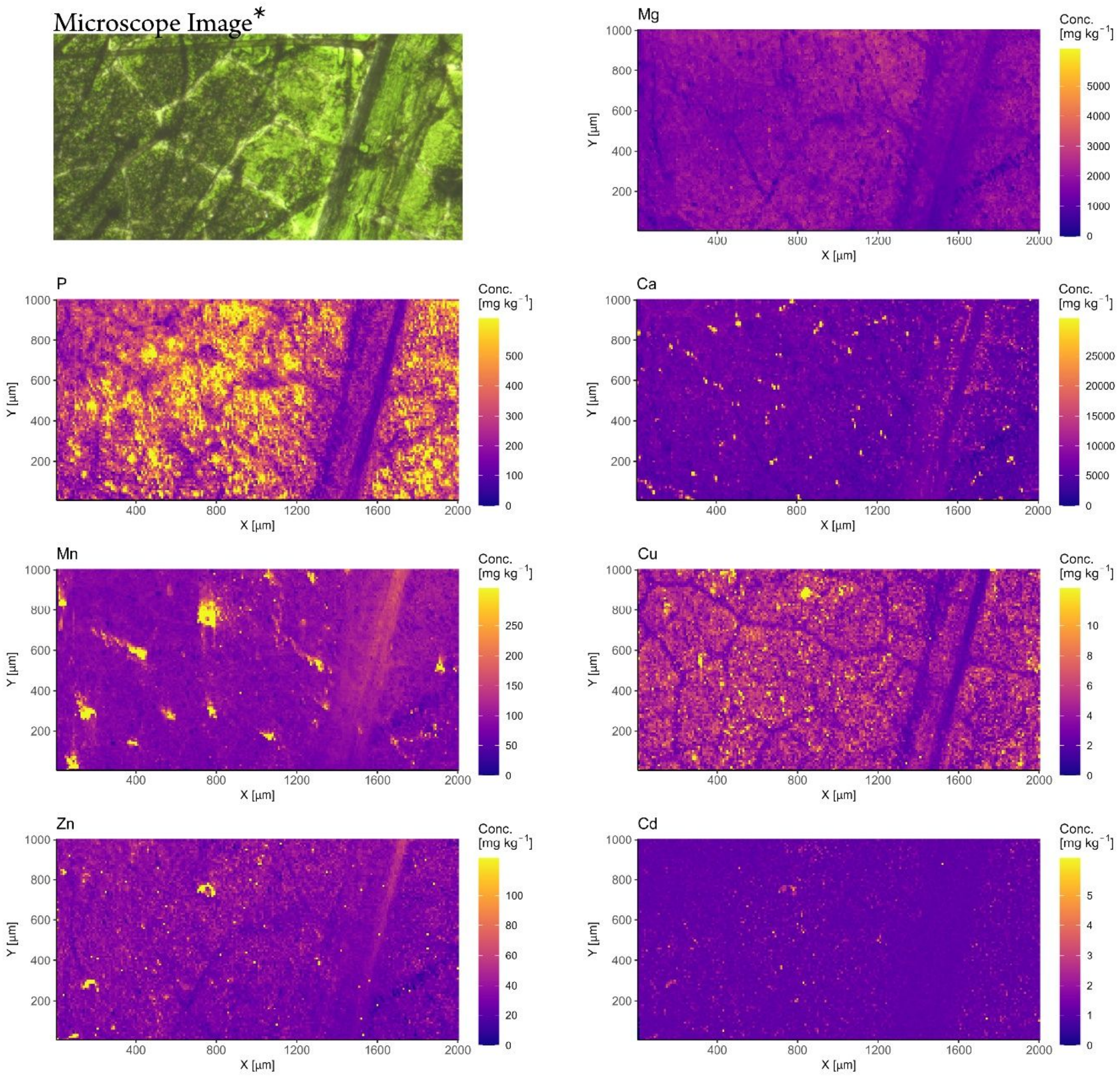

*Figure S4. Element distributions found within a soybean leaf grown on unspiked soil. * Shows the general leaf structure but does not correspond to the measured area.*

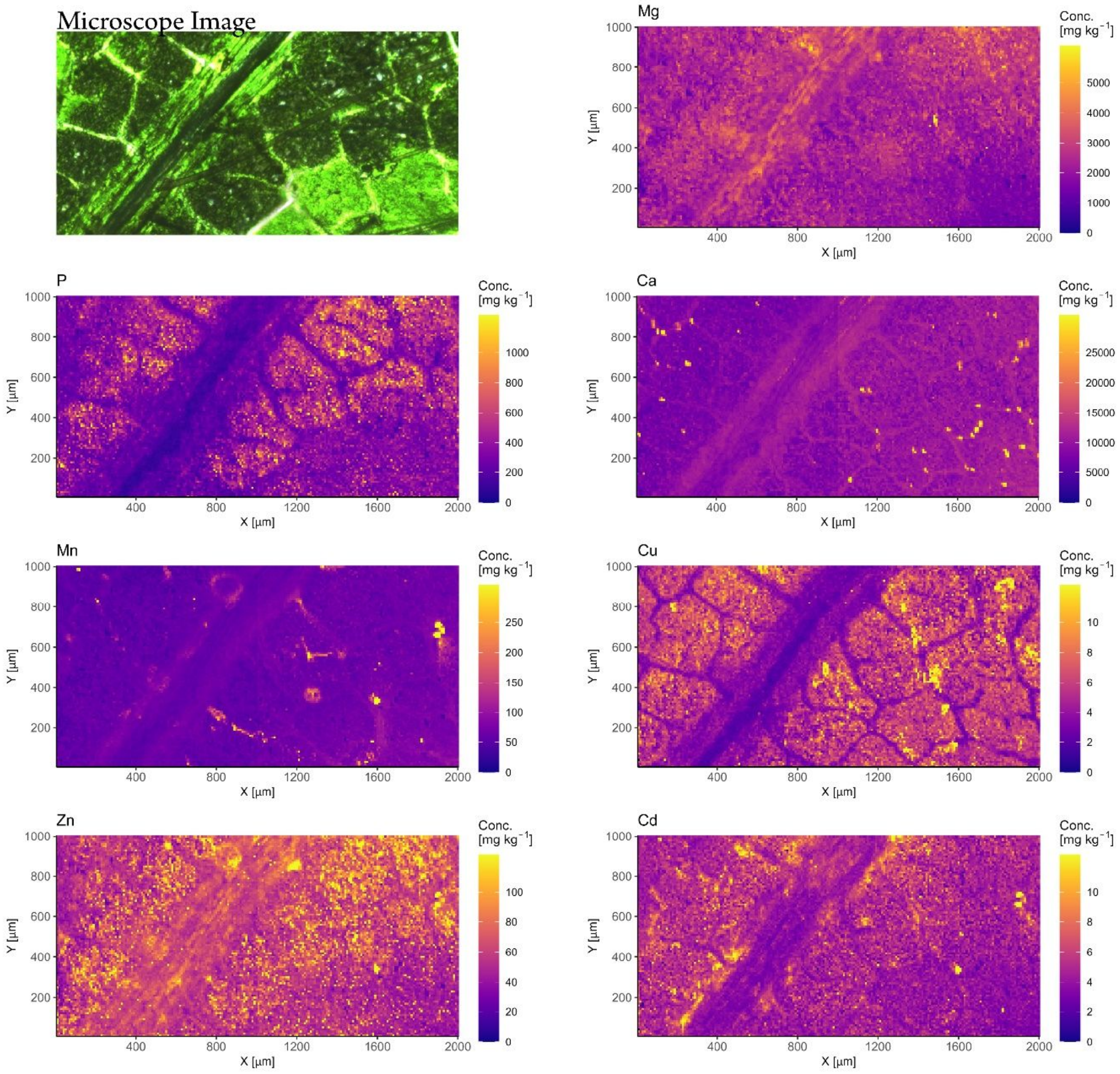

Figure S5. Element distributions found within a soybean leaf grown on Cd-spiked soil.

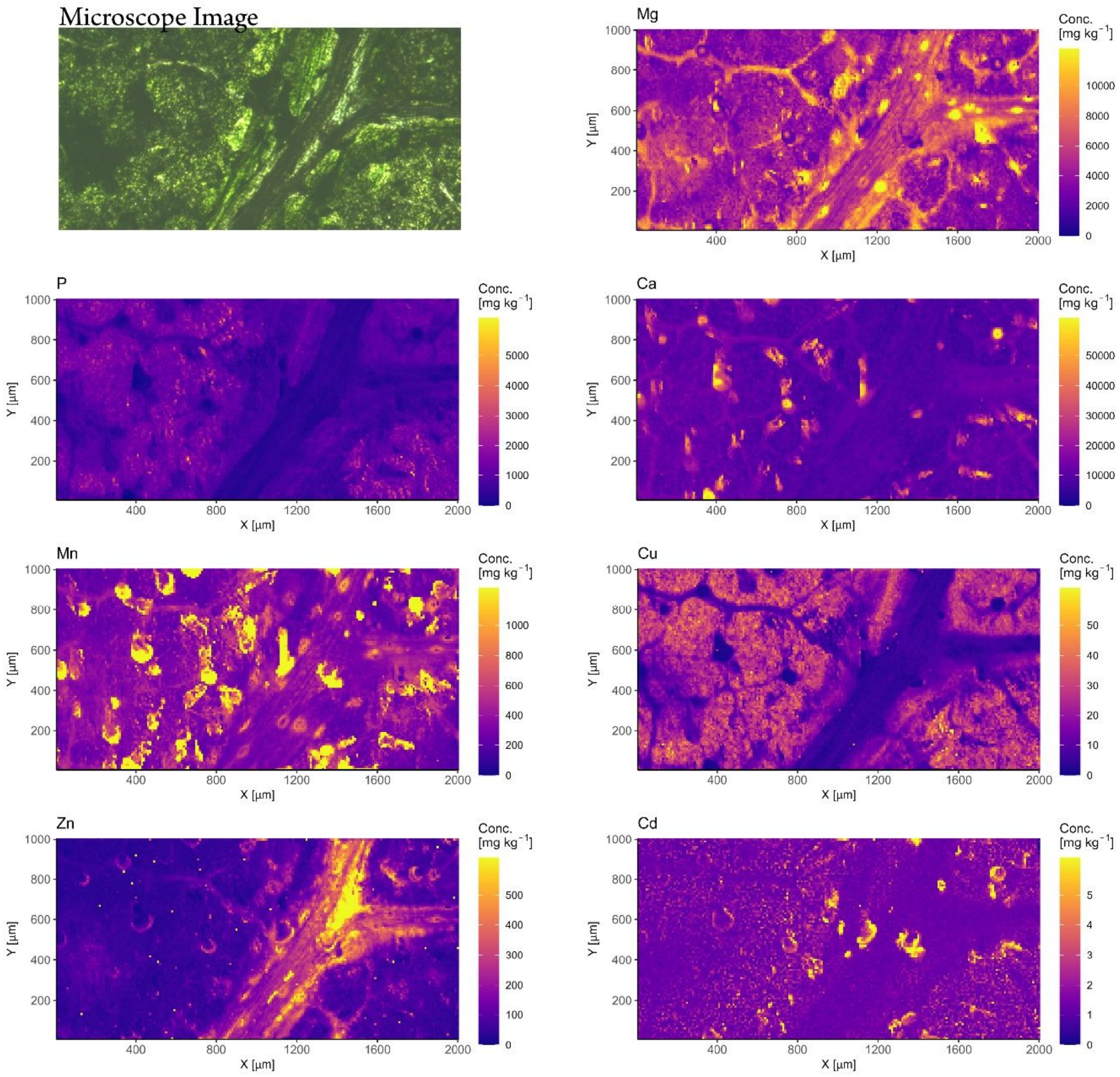

Figure S6. Element distributions found within a sunflower leaf grown on unspiked soil.

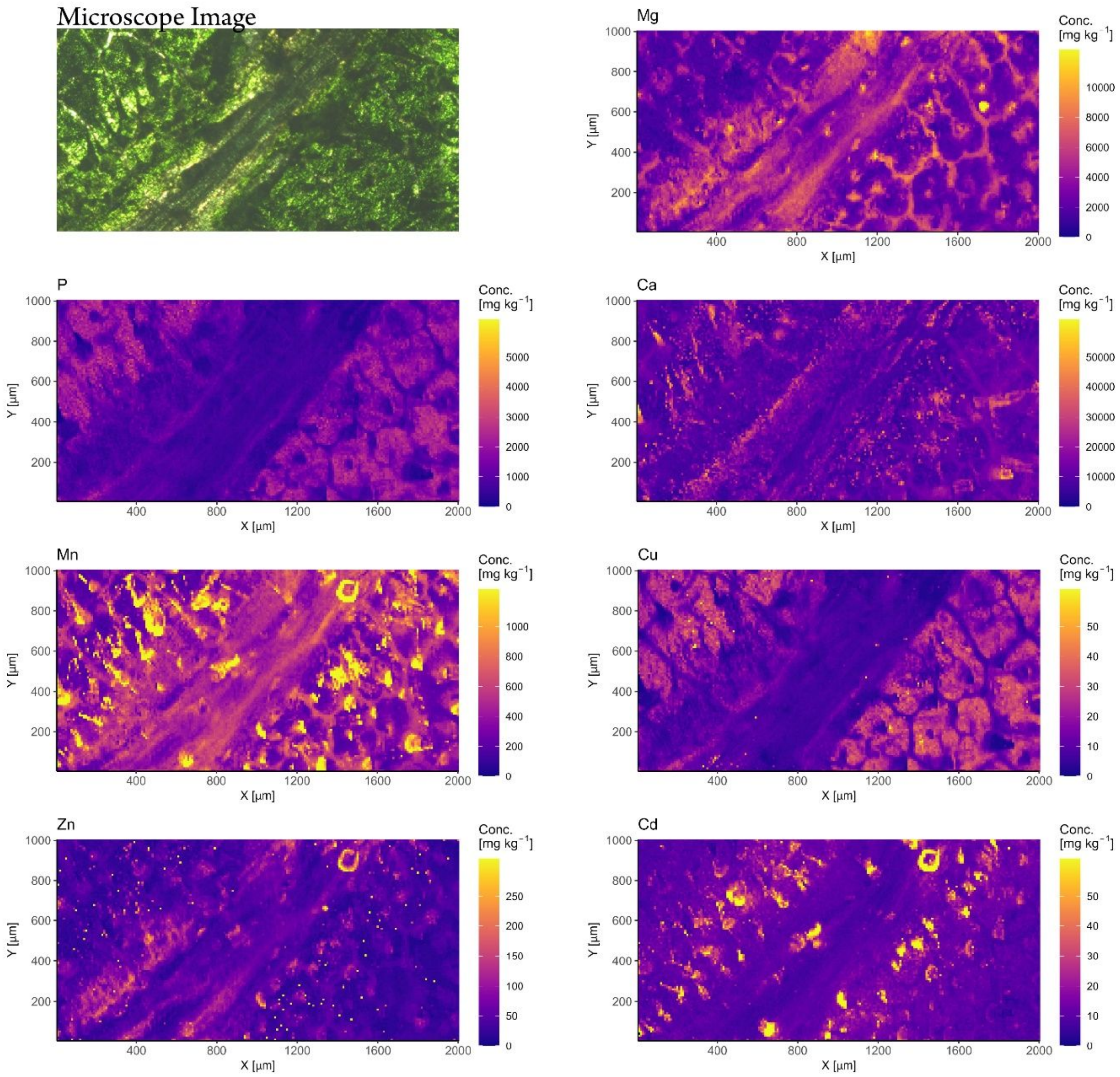

Figure S7. Element distributions found within a sunflower leaf grown on Cd-spiked soil.
